# Phylogenetic detection of horizontal gene transfer during the step-wise genesis of *Mycobacterium tuberculosis*

**DOI:** 10.1186/1471-2148-9-196

**Published:** 2009-08-10

**Authors:** Frédéric Veyrier, Daniel Pletzer, Christine Turenne, Marcel A Behr

**Affiliations:** 1 Department of Medicine, McGill University, Montreal, QC, H3G 1A4, Canada

## Abstract

**Background:**

In the past decade, the availability of complete genome sequence data has greatly facilitated comparative genomic research aimed at addressing genetic variability within species. More recently, analysis across species has become feasible, especially in genera where genome sequencing projects of multiple species have been initiated. To understand the genesis of the pathogen *Mycobacterium tuberculosis *within a genus where the majority of species are harmless environmental organisms, we have used genome sequence data from 16 mycobacteria to look for evidence of horizontal gene transfer (HGT) associated with the emergence of pathogenesis. First, using multi-locus sequence analysis (MLSA) of 20 housekeeping genes across these species, we derived a phylogeny that serves as the basis for HGT assignments. Next, we performed alignment searches for the 3989 proteins of *M. tuberculosis *H37Rv against 15 other mycobacterial genomes, generating a matrix of 59835 comparisons, to look for genetic elements that were uniquely found in *M. tuberculosis *and closely-related pathogenic mycobacteria. To assign when foreign genes were likely acquired, we designed a bioinformatic program called mycoHIT (mycobacterial homologue investigation tool) to analyze these data in conjunction with the MLSA-based phylogeny.

**Results:**

The bioinformatic screen predicted that 137 genes had been acquired by HGT at different phylogenetic strata; these included genes coding for metabolic functions and modification of mycobacterial lipids. For the majority of these genes, corroborating evidence of HGT was obtained, such as presence of phage or plasmid, and an aberrant GC%.

**Conclusion:**

*M. tuberculosis *emerged through vertical inheritance along with the step-wise addition of genes acquired via HGT events, a process that may more generally describe the evolution of other pathogens.

## Background

Bacterial evolution can occur through either the modification of vertically inherited genes or the acquisition of new genes. In the former process, duplication of genes and neo-functionalization represent molecular events for selection to act upon. In the latter process, transformation (acquisition of naked DNA), transduction (acquisition of DNA via a phage infection) and conjugation (acquisition of DNA via a plasmid or a mobile element) represent mechanisms whereby bacteria can incorporate new genes [1]. These modes of acquiring new genetic material are collectively referred to as horizontal gene transfer (HGT) and represent an important means by which an organism can exploit a new condition, such as a pathogenic lifestyle [2,3].

In general, the determination that HGT has occurred is inferred by study at the species level, comparing virulent strains versus others strains within the species. In mycobacteria, gene acquisition has been implicated in the recent and rapid evolution of *M. ulcerans *from *M. marinum *via acquisition of a virulence plasmid [4]. Additionally, *M. avium subsp. paratuberculosis *has acquired strain-specific genetic elements that are not present in the related environmental organism *M. avium subsp. hominissuis *[5,6]. The role of HGT in the recent evolution of *M. tuberculosis *has also been the subject of study, looking at genes in modern *M. tuberculosis *lacking from isolates referred to as *M. prototuberculosis *[7,8]. Unlike the recent evolution, the role of HGT in shaping evolution of microorganisms over a longer time-span, at the genus-level, has received decidedly less attention.

Recently, the availability of genome sequence data for multiple species of a genus has permitted across-species comparisons for gene content. This is particularly pertinent for the genus *Mycobacterium*. The majority of mycobacteria are non-pathogenic environmental organisms, but a minority of species, belonging to the slow-growing sub-lineage, cause important human diseases (*M. tuberculosis*, *M. leprae*, *M. ulcerans*, *M. kansasii*) and veterinary diseases (*M. bovis*, *M. marinum*, *M. avium *subsp. *paratuberculosis*, *M. avium *subsp. *avium*). Because the majority of pathogenic mycobacteria are situated in the same sub-lineage of the genus, we hypothesize that these different species share a common pathogenic potential, upon which selective pressures specifically resulted in the unique attributes of each organism. We further hypothesize that comparisons of all available mycobacterial genome sequences will reveal the degree to which HGT has contributed to 'old' events during the macro-evolution of pathogenic mycobacteria. To test this, we have performed a comprehensive phylogenetic screen, looking for HGT events that have contributed sequentially to the genesis of *M. tuberculosis*. We have also compared these results with findings derived through a compositional analysis to assess for the concordance between these methods [7]. Together, the data present a portrait of the slow, step-wise emergence of virulence, setting the stage for further molecular studies that define the role of each gene acquisition event in the emergence of *M. tuberculosis *as an epidemic pathogen.

## Methods

### Genome sequence data

*M. kansasii *ATCC 12478^T^, *M. avium *subsp. *avium *ATCC 25291^T ^and *M. intracellulare *ATCC 13950^T ^were purchased from the American Type Culture Collection. After growth in Middlebrook 7H9 media (Difco Laboratories, Detroit, MI) containing 0.05% Tween 80 (Sigma-Aldrich, St louis, MO) and 10% of Albumin-Dextrose-Catalase (Becton Dickinson and co., Sparks, MD), genomic DNA was extracted using a protocol optimized for mycobacterial DNA, as previously described [9]. Genomic DNA was used to prepare a library for shotgun sequencing at the University of Minnesota Biomedical Genomics Center (*M. avium *subsp. *avium*) or the McGill University and Genome Quebec Innovation Center (*M. kansasii *and *M. intracellulare*). At both centers, sequences were determined by the Genome Sequencer™ FLX (Roche Applied Science). The genome of *M. kansasii *is estimated to be 6.4 Mb in length, with 33× coverage, and has been deposited in GenBank as projectID number 30907. The genome of *M. avium *subsp *avium *is estimated to be 4.8 Mb in length, with 17× coverage, and is deposited in GenBank as projectID number 30909. The genome of *M. intracellulare *is estimated to be 5.4 Mb in length, with 26× coverage, and is deposited in GenBank as projectID number 27955.

For other genomes, sequences were obtained at NCBI http://www.ncbi.nlm.nih.gov/genomes/lproks.cgi using the parenthesized accession numbers: *M. tuberculosis *H37Rv (NC_000962) [10], *M. marinum *M (NC_010612) [11], *M. ulcerans *Agy99 (NC_008611) [12], *M. leprae *TN (NC_002677) [13], *M. avium *subsp. *hominisuis *104 (NC_008595), *M. avium *subsp. *paratuberculosis *K10 (NC_002944) [14], *M. smegmatis *str. MC2 155 (NC_008596), *M. vanbaalenii *PYR-1 (NC_008726), *M. gilvum *PYR-GCK (NC_009338), *M. sp*. KMS (NC_008705), *M. sp*. MCS (NC_008146), *M. sp*. JLS (NC_009077), *M. abscessus *19977^T ^(NC_010397), *Rhodococcus jostii *RHA1 (NC_008268) [15], *Nocardia farcinica *IFM 10152 (NC_00631) [16] and *Nocardiodes sp*. JS615 (NC_008699).

### Multilocus Sequence Analysis-based phylogeny

Multilocus sequence analysis of 20 house-keeping genes from each mycobacterial genome was performed in order to uncover their phylogenetic relationships. Chosen genes were deemed essential for *in vitro *growth [17] and conserved across all mycobacterial species and the two out-group species. These genes were also present in the markedly decayed genome of *M. leprae*, providing further evidence for their conservation. The genes are distributed throughout the *M. tuberculosis *H37Rv genome: Rv0005-*gyrB*, Rv0041-*leuS*, Rv0350-*dnaK*, Rv0440-*groEL2*, Rv0667-*rpoB*, Rv0684-*fusA1*, Rv0704-*rplB*, Rv1098-*fum*, Rv1310-*atpD*, Rv1437-*pgk*, Rv1650-*pheT*, Rv1832-*gcvB*, Rv2220-*glnA1*, Rv2555c-*alaS*, Rv-2748c-*ftsK*, Rv2839c-*infB*, Rv2916c-*ffh*, Rv3240-*secA1*, Rv3459c-*rpsK*, and Rv3646-*topA*. For an out-group, we used orthologues of these genes in two related members of the order *Actinomycetales*: *Rhodococcus jostii *and *Nocardia farcinica *IFM 10152.

For each gene, representative sequences were obtained and aligned in MEGA 4.1 [18]. Resulting FASTA files were entered and concatenated in START2 [19] to form a final 38787 bp in-frame semantide. Next, using the Neighbor-Joining method, we generated an un-rooted phylogenetic tree at the amino acid level in MEGA 4.1, with 500 bootstrap replicates. Phylogenies were also generated for each individual protein to determine the concordance of findings on a gene-by-gene basis (data not shown). Notably, because the situation of *M. leprae *varied considerably on a gene-by-gene basis, we have removed this organism from the MLSA-based tree.

### Detection of *M. tuberculosis *orthologues in other mycobacterial genomes

To detect *M. tuberculosis *genes that do or do not have orthologues in the other genomes, we performed an alignment search with the StandAlone TBLASTN program [20], using the 3989 predicted proteins from *M. tuberculosis *as the query sequences to search for matches in the genomic DNA of other organisms. With this initial search, we obtained a matrix of 63824 scores (3989 protein sequences blasted against 16 genomes) providing two types of output: categorical (hit versus no hit) and quantitative (degree of similarity). To categorically assign that there was no hit, we employed the default E-value (or Expectation value) of e^-10 ^which is provided at NCBI and has been used in a similar study [21]. Thus, if the statistical significance ascribed to a comparison is greater than this E value, we assigned a percentage of similarity of 0% to that comparison.

To analyze quantitative results, we wished to assign absence of an *M. tuberculosis *orthologue or presence of an *M. tuberculosis *orthologue in the database genome. When no hit was reported (0% similarity), we determined that this *M. tuberculosis *protein has no orthologue in the corresponding database genome. To determine presence of an orthologue, we performed a sensitivity analysis using different thresholds of the similarity score. In the simplest model, any hit was considered evidence for an orthologue (referred hereafter as the 'any hit' threshold). To reduce the likelihood of false-positive assignments due to low-level similarity, we performed an empiric analysis on sequence-comparison data to define incrementally more stringent thresholds. As exemplified in the Additional file 1 (Figure S1), the frequency distribution of BLAST similarity scores obtained between *M. tuberculosis *and *M. kansasii *was non-Gaussian, with a right skew. Therefore, we did not use thresholds guided by the mean and the standard deviation. Rather, we determined the degree of similarity at the 95^th ^percentile of hits between the organisms under comparison, then at the 90^th ^percentile and finally at the 85^th ^percentile. As the similarity score that met these percentiles varied across different genome comparisons, we determined these thresholds for each bi-organism comparison before applying these cut-offs to assign orthology.

### Phylogeny-based detection of horizontal gene transfer in *M. tuberculosis*

To enumerate genes potentially acquired via horizontal gene transfer, we looked for proteins found only in certain subsets of the MLSA-based phylogeny. We assumed that a protein encoded by a foreign gene will be present in at least some of the descendants of the bacteria that acquired it, but will be absent from other closely related bacteria. As an example, for a *M. tuberculosis *protein with orthologues in *M. kansasii*, *M. marinum *and *M. ulcerans*, but no orthologue in other mycobacterial sequences, we inferred that this gene was potentially acquired by the common ancestor of *M. tuberculosis*, *M. kansasii*, *M. marinum *and *M. ulcerans*. The alternative hypothesis would be that this gene was present in the ancestral *Actinobacteria *but lost independently three times (from *M. avium-intracellulare*, from rapid-growing mycobacteria, and from *Rhodococcus jostii*). This alternative hypothesis was further considered in a secondary analysis described below under 'sub-classification analysis.'

**Figure 1:**
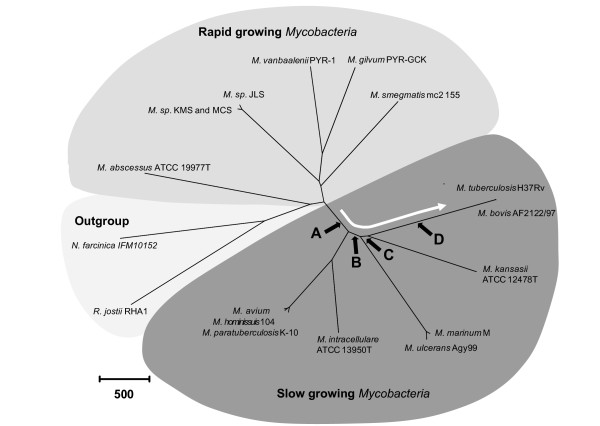
**Phylogeny of *Mycobacterium *genus and schematic representation of acquisition of foreign genes at different strata of *M. tuberculosis *evolution**. The radial tree was generated using MEGA 4.1. The different mycobacterial lineages, based on slow-growing versus rapid-growing organisms, are also indicated. The arrow indicates schematically the positions of the different list of HGT, detected in this study (A, B, C and D), during the step-wise evolution of slow-growing mycobacteria. The scale represents the number of amino acid differences.

Therefore, using the MLSA-based phylogeny, we assigned gene acquisitions into four lists. List A included genes acquired by the ancestor of all slow-growing mycobacteria, potentially including elements mediating common pathogenicity mechanisms across these different organisms. List B included genes acquired by the common ancestor of *M. tuberculosis*, *M. kansasii*, *M. marinum *and *M. ulcerans*. List C included genes acquired by the common ancestor of *M. tuberculosis *and *M. kansasii*. List D included genes unique to *M. tuberculosis*, acquired after the common ancestor with *M. kansasii *(see Figure 1).

To facilitate the process of assembling the matrices of similarities into potential HGT assignments, we developed a software utility called mycoHIT (mycobacterial Homology Investigation Tool). The Software and a help file are available at http://www.molepi.mcgill.ca. Briefly, mycoHIT is a JAVA application presented through a graphical user interface. This program serves to perform BLAST searches, parses the TBLASTN result files with PERL scripts, in order to select the highest score for each protein. Obtained data, including the score, are then stored on a local MYSQL database. Our goal was to define proteins for which an orthologue was present in one group of organisms (e.g. *M. kansasii, M. marinum, M. ulcerans*) but for which there was no orthologue across all of the remaining organisms (e.g. *M. avium-intracellulare*, rapid growing mycobacteria and *Rhodococcus *jostii). Because this analysis was based on these two criteria of orthology (present in some, absent from the rest), we were forced to exclude *M. leprae *from this analysis because the *M. leprae *genome is characterized by pseudogenes and deletions [13] which resulted in an unacceptable degree of error in categorization of orthologues and an uncertain phylogenetic situation.

To focus our analysis on HGT during the evolution of slow growing mycobacteria, we selected these 7 genomes (*M. kansasii, M. marinum, M. ulcerans, M. avium *subsp. *hominissuis, M. avium *subsp. *paratuberculosis, M. avium *subps. *avium *and *M. intracellulare*) and the option "compute all combinations for selection" (see Additional file 1 Figure S2-A). Given the selection of these 7 comparisons, the program generated 128 files (2^7^), representing the lists of *M. tuberculosis *genes that have orthologues in 0 to 7 slow growing mycobacterial genomes and lack orthologues in the remaining 9 to 15 genomes (i.e. proteins with orthologues in *n *genomes but lacking orthologues in (15- *n*) genomes). These lists provide the number of genes differentially shared across slow-growing mycobacteria in each of the 128 possible combinations. Subsequently, the program calculates "special lists", representing the chosen sum of the previously calculated 128 lists according to the phylogeny (See additional file 1 – Figure S2-B). These lists are referred to as lists A, B, C, D. As an example, list B will be the sum of lists MM (orthologue in *M. marinum *absent in all other), MU (orthologue *M. ulcerans *absent in all other), MM-MK (orthologues in *M. marinum*, *M. kansasii *absent in all other), MM-MU, MU-MK and MM-MU-MK.

The mycoHIT program was intensively tested to report potential problems. Furthermore, to ensure that the program sorted genes correctly, we manually performed the analysis twice using two different thresholds ("any hits" and 60% identity). Briefly, using EXCEL we sorted genes and excluded those with hits in at least one of the rapid growing species or *R. jostii*. In a second phase, we manually grouped genes into lists A, B, C and D following the same strategy explained above. As mycoHIT generated the same genes and numbers at these two thresholds as we obtained in the manual search, this comparison served to validate the mycoHIT program.

### Sub-classification and characterization of potential HGT

For further analysis, we began with the most inclusive list obtained through the least restrictive threshold ("any hit") to avoid the risk of overlooking potentially instructive HGT events. For all these genes, we performed a blastP of the predicted encoded protein using the NCBI database composed of 853 bacterial genomes (including 53 *Actinobacteria*) in order to classify the proteins into four categories 1) proteins with no blast hits at all in the entire NCBI database, potentially indicating false-annotation, 2) proteins that may not represent HGT (putative deletions), based on presence of homologues in at least two different genera of *Actinobacteria *out of the first five taxonomic classes hits, 3) proteins that provide evidence for HGT but represent vehicles of HGT, such as transposons, phage, toxin-antitoxin genes, and 4) proteins with hits in the NCBI database but one or less hit in the *Actinobacteria *class out of the first five taxonomic classes presenting hits, most likely to represent the bacterial elements acquired through HGT. Notably, with this approach, we expect that proteins that are part of a conserved family (e.g. ABC transporters) will never be assigned as HGT, due to low- similarity hits. We partially correct for this possibility when such a gene was situated in a HGT cluster. To this end, when a cluster of HGT genes was detected, internal genes not detected through the phylogenetic analysis were added if they met the aforementioned criteria (presence of hits in the NCBI database but one or less hit in *Actinobacteria*).

For further analysis, we have calculated the proportion of predicted bacterial HGT associated with an annotated vehicle of HGT (transposons, phage, toxin-antitoxin genes) within 5 genes upstream or downstream of the predicted HGT gene or cluster. We enumerated the number of genes that were located in clusters of two or more putative HGT genes. Schematic representation of GC content across a given region was obtained using Bio-Annotator from VectorNTI (Invitrogen) using 2000 bp windows. Finally, we compared this list of genes with already-published genomic databases on *M. tuberculosis*, specifically: a) essential genes for survival *in vitro *[17], in macrophages [22] and in mice [23], and b) dispensable genes as shown by their deletion in clinical isolates [24] c) genes presenting atypical compositional characteristics suggestive of HGT [7].

## Results

### MultiLocus Sequence Analysis-based phylogeny

Analysis of 20 genes across 16 mycobacterial species, *Rhodococcus jostii *and *Nocardia farcinica *revealed a total of 12741 polymorphic sites at the protein level. The number of amino acid differences between mycobacteria strains ranged from 0 (between *Mycobacterium *sp. KMS and MCS) to 4166 (between *M. bovis *and *R. jostii*). The numbers of amino acid differences are tabulated in the Additional file 1 (Figure S3). An un-rooted phylogenetic representation of these differences, using MEGA 4.1, is shown in Figure 1. This phylogeny presents three groups of organisms: the out-group bacteria, the rapid-growing mycobacteria and the slow-growing mycobacteria. Within the latter group, there were four major lineages: 1) *M. avium*-*intracellulare*, 2) *M. marinum-ulcerans*, 3) *M. kansasii*, and 4) *M. tuberculosis *and related members of the *M. tuberculosis *complex. Individual trees based on the selected genes also presented a similar phylogenetic portrait of the genus (data not shown). Notably, the unexpected finding that *M. kansasii *was the nearest neighbor to the *M. tuberculosis *complex was supported in the majority of single gene phylogenies.

### Genes acquired by HGT in *M. tuberculosis *detected by a phylogenetic method

We aimed to describe not only the HGTs specific to *M. tuberculosis *but also those acquired at each node of separation in the phylogeny (excluding *M. leprae*). Using the different thresholds for orthology, we obtained lists of *M. tuberculosis *proteins where an orthologues is present in 0 to 7 slow growing mycobacteria but absent in all other genomes. As seen in Figure 1, in conjunction with the MLSA-based analysis, we have grouped these proteins into the following lists: List A, genes likely acquired by the ancestor of all slow growing mycobacteria, List B, genes likely acquired by the ancestor of the *M. marinum*-*ulcerans*/*M. kansasii*/*M. tuberculosis *lineage, List C, genes likely acquired by the ancestor of the *M. kansasii*/*M. tuberculosis *lineage, and List D, genes likely acquired in the *M. tuberculosis *lineage. To test for the robustness of these List assignments, we looked for evidence of genes that are discordant with the proposed phylogeny. To test the MLSA-based prediction that *M. kansasii *is the nearest neighbor among sequenced strains to *M. tuberculosis*, we determined how many genes are shared between *M. tuberculosis *and *M. kansasii*, but no other species, as compared to the number of genes that are shared only between *M. tuberculosis *and *M. marinum*. Under a null model, the two comparisons (*M. tuberculosis*/*M. kansasii *versus *M. tuberculosis*/*M. marinum*) would have an equal number of orthologues. This hypothesis was rejected by observing that there were a total of 72 such orthologous pairs; 42 in the case of *M. tuberculosis*/*M. kansasii *compared to 30 in the case of *M. tuberculosis*/*M. marinum*. (p < 0.05 by exact Binomial method). Therefore, as orthology searches provided independent support for the MLSA-based phylogeny, we proceeded to enumerate acquired genes at different steps in the evolution of *M. tuberculosi*s (Table 1). When using different thresholds of orthology ("any hit", 95^th ^percentile, 90^th ^percentile or 85^th ^percentile), we obtained slight shifts in these numbers, but overall a stable estimate of around 11–14% of the genome was assigned as potential HGT (versus *predicted *HGT, based on more stringent criteria, as described below). Notably, about one half of these were specific to *M. tuberculosis *(List D).

**Table 1:** Numbers of genes detected as potential HGTs using different parameters:

Thresholds	List A	List B	List C	List D	Total
"any hits" (> 1, < 0)	142	90	42	274	548
95 percentile (48,53,53)	134	83	41	274	532
90% percentile (56,61,61)	112	74	39	274	499
85% percentile (66,66,66)	87	65	31	274	457

In addition to helping uncover HGT, the mycoHIT program can also be used to detect potential deletion events during the step-wise genesis of *M. tuberculosis*. This approach, along with associated lists, are provided in Additional file 2. Of note, list Dd comprises 689 genes potentially deleted from the ancestor of *M. tuberculosis *after its separation with *M. kansasii*. This list is approximately three times longer than the corresponding list D of potencial HGT. These results emphasize that in parallel with the acquisition of foreign genes, there has been reduction of the *M. tuberculosis *genome associated with pathogenic specialization, as has been described for other mycobacterial species [6,12]. Unlike *M. marinum *and *M. kansasii*, *M. tuberculosis *has not been isolated from the environment, therefore the process of reductive genomics was potentially due to lack of selective pressure to retain genes important for environmental survival.

### Sub-classification of potential HGT

**Figure 2:**
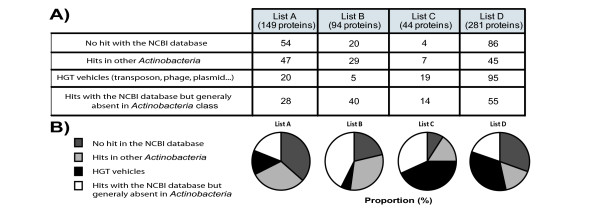
**Sub-classification of potential HGT**. **A) **Number of proteins from the different lists (A, B, C and D) presenting the specified characteristics. The lists were obtained using the "any hits" threshold in mycoHIT, each predicted protein was blasted against the NCBI database, and the results were used to classify them in the specified category as described in material and methods. **B) **Proportion of proteins, from list A, B, C and D, which present the specified characteristics.

To determine genes most likely to represent true-positive HGT, we analyzed the total list of genes obtained with the "any hits" threshold to find those that were unlikely to represent HGT, either because they do not code for a protein or because the encoded protein has multiple orthologues in other genera of *Actinobacteria*. Figure 2 presents the number (2A) and the proportion (2B) of genes from the four lists in each category. The first category represents genes with no blast results across the NCBI database, suggesting that these genetic sequences do not code for a protein product or instead that the encoded proteins are so small that they cannot meet the minimal E-value for an orthologue. This situation applied to 36% of List A proteins, 21% of list B proteins, 9% of list C proteins and 30% of list D proteins. These results were not unexpected. For instance, the high proportion in list A is explained by duplication of small proteins (e.g. 56% of proteins in this category are Esx and PE proteins).

The next category of potentially false-positive HGT genes was those for which we observed orthologues in at least two different genera from the *Actinobacteria *phylum. Although some of these genes could represent HGT events within the family or multiple transfers, to be conservative, we assumed that these may represent ancestral genes that have been deleted in certain lineages, resulting in a restricted distribution (e.g. deleted from the rapid-growing mycobacteria lineage and *Rhodococcus jostii*. but present in all slow-growing mycobacteria). The proportion of proteins classified in this category represents 31%, 31%, 16% and 16% respectively from lists A, B, C and D.

Of genes most likely to represent HGT, we divided these between the process of HGT and the accompanying bacterial genes. Genes with homology to transposons, phage and toxin-antitoxin comprised 13% of proteins from the list A, 5% of proteins from list B, 43% of proteins from list C and 34% of proteins from list D. The lower proportion of HGT vehicles in the earlier lists is consistent with the expectation that these genes may be eliminated with greater time due to a lack of selective pressure for their maintenance.

Finally, the remaining proteins (listed in table 2) on each of Lists A-D were considered to represent bacterial genes acquired through the process of HGT. Based on these Lists, we obtained 28 *M. tuberculosis *genes that were acquired by the ancestor of all slow growing bacteria, 40 that were acquired by the ancestor of the *M. marinum*-*ulcerans*/*M. kansasii*/*M. tuberculosis *lineage, 14 that were acquired by the ancestor of the *M. kansasii*/*M. tuberculosis *lineage and finally 55 that were specifically acquired by the ancestor of the *M. tuberculosis *complex. Together, this makes a most conservative estimate that in addition to vehicles of HGT, 137 genes (28 + 40 + 14 + 55) in *M. tuberculosis *were acquired through the process of HGT. As a note, we also performed a PSIBLAST (Position-Specific iterated BLAST) on the 55 proteins from ListD (data not shown). Only two proteins (both regulators) have been found to have a hit in other *Actinobacteria*. This argues that the HGT estimate is not biased by high sequence variability within a vertical transmitted protein.

**Table 2:** List of genes detected, with high confidence, to be horizontally transferred:

Name of Gene	Annotation (essential genes)	Gene GC% (*M. tb *= 65%)	Presence of HGT vehicle (Yes or No)
**List A**			
Rv0082 to Rv0087	*hyc *locus (82 *ex vivo*; 85–86 *in vitro*)	69.1	N
Rv0113 to Rv0115	sedoheptulose-7-phosphate isomerase	61.3	N
Rv1006	Glycosyl transferase	65	N
Rv1513/14c, Rv1516c, Rv1518 and Rv1520	LOS locus (14c: *in vivo*)	NA	Y
Rv1722	Carboxylase	67	Y
Rv2067c	Methyltransferase	59.5	Y
Rv2387	Permease (*in vivo*)	61.8	N
Rv2531	Arginine Decarboxylase	63.4	Y
Rv2561/62	H.P	59.9	N
Rv2633c	Hemerythrin HHE cation binding domain scavenger	60.8	N
Rv2955c to Rv2957 and Rv2963	PGL locus	NA	Y
Rv3528c	Methyltransferase	48.6	N
Rv3768	Permease	61.8	Y
Rv3788	Transcription Elongation GreA	66.3	N
**List B**			
Rv0104	cAMP-kinase regulatory subunit	63.8	N
Rv0193c	H.P.	61.6	N
Rv0213c/14c	Methyltransferase and NadR	63.4	N
Rv0347	H.P (*in vitro*)	62.1	N
Rv0379	SecE2	63.4	N
Rv0520/21	Methyltransferase	62.1	N
Rv0793	Antibiotique monooxygenase	66.3	Y
Rv0899	OmpA	60.8	N
Rv1192	PGAP1 family (*in vivo*)	67.8	N
Rv1289	H.P	60.0	N
Rv1371 to Rv1376	Fatty acid desaturase, Pks18, glycolipid sulfotransferase, TfuA like protein (71: *in vivo*)	63.4	Y
Rv1500 to Rv1508c and Rv1525	LOS locus	NA	Y
Rv1541c	H.P.	63.5	N
Rv1732	Thioredoxin	69.6	N
Rv1749	H.P.	62.2	N
Rv1995	Hemerythrin HHE cation binding domain scavenger	63	Y
Rv2075c	C-type lectin domain	68.3	N
Rv2277	Glycerophosphodiesterase (GdpD) (*in vivo*)	61.9	Y
Rv2289	CDP-diacylgylcerol pyrophosphatase	57.5	N
Rv2636	chloramphenicol 3-O-phosphotransferase	63.4	N
Rv2761c	type I restriction/modification system	63.7	Y
Rv2949c	PGL locus: 4-hydroxybenzoate synthetase	52.5	Y
Rv2958c and Rv2962c	PGL locus: glycosyltransferase	64.8 and 65.8	Y
Rv3091	patatin-like phospholipase family protein	69.4	N
Rv3172c	H.P.	59.6	N
**List C**			
Rv0325/26	Methyltransferase (26: *in vivo*)	63.9	N
Rv0611c	H.P.	63.9	Y
Rv0628c and Rv0874c	H.P. (28c: *in vitro*)	72.2 and 70.5	Y and N
Rv1498c	Los locus: Methyltransferase	58.7	Y
Rv1671	H.P.	62.1	N
Rv2292c/93c	Methylthioadenosine nucleosidase	63.9	N
Rv2959c	PGL Locus: Methyltransferase	53.7	Y
Rv3081	H.P.	64.8	N
Rv3138	Pyruvate formate lyase activating enzyme	61.9	N
Rv3373/74/75	Enoyl-CoA Hydratase 18, Amidase (75: *in vivo*)	59.7	Y
**List D**			
Rv0032/33	BioF/AcpA	61.6	Y
Rv0059/60	Appr1-P (60: *in vitro*)	60.8	Y
Rv0078A	H.P.	61.1	N
Rv0329c/30c	Methyltransferase, TetR	67.5	N
Rv0987/88	Adhesion/permease and hydroxymethylcoA reducatase	53.9	N
Rv1045 to Rv1049	H.P. (45 and 49: *ex vivo*)	64.6	Y
Rv1509 and Rv1515c	LOS locus: Methyltransferase	NA	Y
Rv1552 to Rv1555	Fumarate reductase	63.4	Y
Rv1673c/74c	transglutaminase-like/ArsR	64	N
Rv2003c, Rv2008c and Rv2011c	Methyltransferase/ATPase, AAA+/H.P. GI4	NA	Y
Rv2295	H.P.	64.5	N
Rv2336/37c/38c	Molybdoterine thyamine synthesis (38c: *in vitro*)	58.8	N
Rv2432c/33c	H.P.	63.8	N
Rv2491/92	H.P.	54.8	Y
Rv2735	H.P.	56.5	Y
Rv2804c and Rv2816c to Rv2826c	H.P. GI6 (17c: *in vitro*)	62.1	Y
Rv2954c	PGL locus: Methyltransferase	62.8	Y
Rv2990c	Methyltransferase	62.5	N
Rv3122/23	H.P.	67.8	N
Rv3189/90c	filamentous haemagglutinin-adhesin/H.P.	62.0	Y
Rv3376/77c/78c	Production of the halimane skeleton (76c: *ex vivo*)	54.7	Y
Rv3402c/03c/04c	Aminotransferase/FAD dependent oxidoreductase/formyltransferase	62.6	Y
Rv3471	Cupin family protein	65.9	Y

Neighboring genes were abbreviated using the last two numbers and the letter c (if required) for the second or third genes (e.g. Rv1513 and Rv1514c become Rv1513/14c). NA = Not Applicable (GC% not presented for sets of separated genes), H.P. = Hypothetical protein, GI = Giant island (see Additional file 1 – Figure S4). Genes previously described as essential for *M. tuberculosis *by transposon screens [17,22,23] are indicated in parentheses after the annotation.

### Predicted HGT reveal characteristics other than abnormal phylogeny

**Figure 3:**
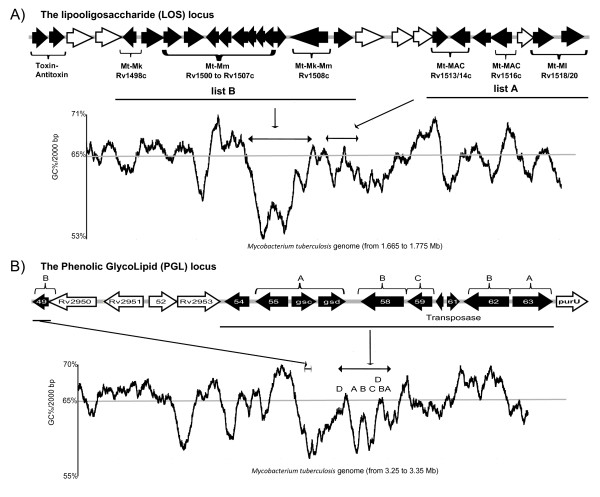
**Example of lineage specific biochemical properties mediated via the acquisition of foreign genes**. **A) **The lipooligosaccharide (LOS) locus is represented from Rv1494 to Rv1520. A graphical representation of the GC content, across a 110 Kb DNA sequence, illustrates the GC% drop of the DNA comprising the indicated clusters of HGT. **B) **The phenolic glycolipid (PGL) locus is represented from Rv2949c to Rv2964. Genes colored in black are those detect as HGT. Again, a graphical representation of the GC content, across a 100 Kb DNA sequence, illustrates the GC% drop of the foreign DNA. For visual reasons, some small genes have been labeled with the two last numbers (e.g. Rv2949c has been named 49c). In all case, the genes colored in black are those detect as HGT (or HGT vehicle), and unless otherwise stated, genes are from list D.

**Figure 4:**
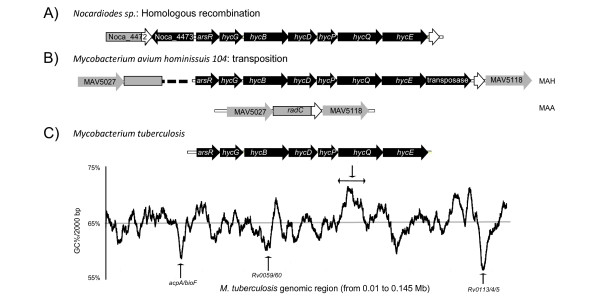
**Example of anaerobic adaptation via the acquisition of a locus transferred several times in *Actinobacteria***. A) Representation of the formate hydrogenase locus (*hyc*) locus in *Nocardiodes sp*.. A part of the Noca_4472 gene (*pckA*) has been duplicated at each side of the *hyc *locus, suggesting integration of the *hyc *locus by homologous recombination. B) Representation of the *hyc *locus in *M. avium *subsp. *hominissuis *compared with the corresponding region in the *M. avium *subsp. *avium *genome. The presence of a transposase and the split of *radC *into two pieces suggests integration by transposition of the *hyc *locus in *M. avium *subsp. *hominissuis*. **C) **Representation of the hyc locus in *M. tuberculosis *and the corresponding GC% increase. The graphical representation of the GC content, across a 135 Kb DNA sequence, illustrates other HGT elements with GC% decrease, as indicated.

**Figure 5:**
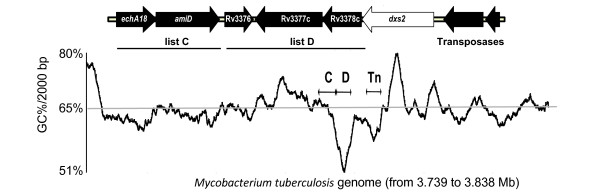
**Example of a virulence locus, not yet functionally characterized, acquired via horizontal genes transfer**. The Rv3375 locus is represented from Rv3374 to Rv3381c, genes colored in black are those detect as HGT (or HGT vehicle). A graphical representation of the GC content, across a 100 Kb DNA sequence, illustrates the GC% drop of the DNA comprising the indicated clusters of HGT and accompanying transposases.

When looking at the location of HGT in the genome, we observed that bacterial genes acquired horizontally appeared in clusters, often located in the same region as an HGT vehicle (see Additional file 1 – Figure S4). 99 of the 137 genes (72%) were associated with another horizontally acquired gene, with the highest proportion seen in list D (87%). Of the 137 genes, 82 (60%) were associated with a HGT vehicle, with the highest proportion again observed with list D (76%). To illustrate compositional signs of HGT (presence of vehicle, clustering or skewed GC%), we graphically present four examples: the lipo-ligosaccharide (LOS) locus (Figure 3A), the phenolic glycolipid (PGL) locus (Figure 3B), the *hyc *locus (Figure 4) and the *Rv3775 *locus (Figure 5). These examples were chosen for their pertinence in the *M. tuberculosis *virulence as discussed below.

For the LOS locus, one observes potential vehicles of HGT (Toxin-Antitoxin genes), two clusters (one a set derived from List B, the other comprising mostly List A genes) and altered GC%. For the List A cluster, the GC% was near 65%, which is the average GC% for the *M. tuberculosis *genome. In contrast, the cluster of List B genes was associated with a drop in GC%, as low as 53% over a calculated interval of 3000 bp.

In the PGL locus, we detect transposases and again these genes are in clusters. List A genes were found at each extremity, whereas genes acquired more recently (Lists B-D) were detected in a more central situation. The GC content of these gene clusters was associated with their assigned list; genes from Lists D and B had no GC% skew whereas genes from lists C and A had decreased GC%.

The third example is the *hyc *operon (Figure 4). Among *Actinobacteria*, the *hyc *locus is found in three distinct forms. In *Nocardiodes sp*. the *hyc *locus features neither a transposase nor a split gene (Figure 4A). Rather, one observes at each side of the *hyc *locus a duplication of a part of a conserved gene (Noca_4472), suggestive of insertion by homologous recombination. In *M*. *avium*, this locus is part of a large island, called LSP^A^6 [25], that is found in *M. avium *subsp. *hominissuis *but not other *M. avium *subspecies. This island includes one transposase and has split *radC *into two pieces, suggestive of insertion by transposition (Figure 4B). Finally, there is a *hyc *locus in *M. tuberculosis*, *M. kansasii*, *M. marinum *and *M. ulcerans *without features of transposition or homologous recombination. Nonetheless, this locus has increased GC content, in contrast to other HGTs in the same region (*acpA/bioF*, Rv0059/60, Rv0113/14/15) where GC% is decreased (Figure 4C). These three variants of the *hyc *locus suggest transfer across bacteria and provide mechanistic clues regarding the mode of acquisition in two of three lineages.

The last example presented in Figure 5 is the Rv3375 locus, which comprises the insertion of two clusters of foreign genes (two genes acquired in list C and three genes acquired in List D). Again, there are transposase elements close to the locus and a GC% drop depending on which list the genes derived from: genes from List C are not associated with an evident GC % change, unlike the decrease seen for List D genes.

A recent study looked for HGT in *M. tuberculosis *using altered GC% at the first and third codon positions or the codon usage as the search criteria [7]. We note that 48% of genes reported in this paper were also detected in our study. More interestingly, 30% of the HGT detected in our study were also detected in this other report, with a higher proportion among our List D genes (57%). This enrichment of compositional features of HGT in list D (the most evolutionarily recent) is expected, as characteristics of HGT tend to homogenize with the rest of the genome and dispensable HGT (e.g. phage proteins) tend to be deleted with time [26]. Moreover, these findings highlight the complementarity of the two approaches. While compositional methods may predict certain HGT that would be overlooked by phylogenetic methods (e.g. between bacteria of the same phylum), the use of our phylogenetic approach has permitted detection of instances of HGT where the genomic signature is unremarkable based on compositional considerations, such as more ancestral events.

### Role of HGT in the emergence of *M. tuberculosis*

Upon analysis of the predicted function of the 137 proteins, an evident increase in transferases (e.g. glycosyl-, formyl-, aminotransferases) was observed (30/137 = 22%). These enzymes transfer a functional group (e.g. methyl) from one molecule to another. Two regions that have been particularly affected by acquisition of foreign transferases are the LOS locus and the PGL locus. Apart from transferases, the HGT lists also contain genes coding for proteins involved in metabolic functions related to adaptation to anaerobic conditions. We detected two such regions: the *hyc *(formate hydrogenlyase operon) and the *frd *(fumarate reductase operon).

Given the evidence in favor of HGT, we evaluated the genes so acquired for clues regarding their potential role in the genesis of *M. tuberculosis *as a specialized pathogen. By comparing the 137 genes against available databases, we assessed for whether these genes were predicted to be essential for growth *in vitro *[17], in macrophages [22], or during experimental *in vivo *infection using the mouse model [23]. As indicated in Additional file 1 (Figure S5) and highlighted in Table 2, we found that 18 genes out of 137 were deemed essential by one of these screens, some clustered in the same region (e.g. two genes essential *in vivo *were in the Rv3375 locus whereas the *hyc *operon contained two genes essential *in vitro *and 1 gene essential during macrophage infection). We also compared against genes deleted in clinical isolates of *M. tuberculosis *[24] as evidence of their dispensability for full virulence in the human host. In this case, 21 of the 137 genes (primarily from list B) have been deleted in at least one clinical isolate. These results suggest that while some genes acquired by horizontal transfer are necessary for full virulence, others may represent genes that were important at earlier stages in the evolution of slow-growing mycobacterial pathogens but are apparently dispensable for the specific pathogenicity of *M. tuberculosis*.

## Discussion

Previous genomic study on *M. tuberculosis*, using sequence level comparisons and post-genomic analysis, has shown that this organism has evolved through clonal evolution, mutation and gene deletion [27,28]. Importantly, these studies have largely concentrated on within-species variability, and therefore have emphasized devolution once an organism is already *M. tuberculosis*. Our study indicates that ancestors of *M. tuberculosis *complex organisms acquired at least 137 coding genes in a step-wise manner through the process of HGT. More specifically, the emergence of the epidemic pathogen, *M. tuberculosis*, from an environmental ancestor has coincided with 'infection' by phages and/or transposable elements after the common ancestor with *M. kansasii*. This scenario is supported by the clinical and epidemiologic observations that *M. kansasii *can cause TB-like pulmonary disease, without evidence of person-to-person spread.

Interestingly, we have detected certain 'hotspots' for HGT, where one observes genes from different lists, indicative of distinct HGT events. This may be due to the presence at these sites of DNA sequences with characteristics (e.g. transposase insertion sequence, homologous DNA to phage DNA) that permit independent HGT. Alternatively, features acquired in a first HGT event may permit secondary insertions due to sequence similarity with DNA of a potential donor. In two 'hotspots', acquisition of a high number of transferases is clearly linked to lineage-specific biochemical properties. Phenolic glycolipids (PGLs) are synthesized on the backbone phthiocerol dimycocerosate (DIM), which is common to all mycobacteria, by adding a phenol ring and methylated sugars (reviewed in [29]). PGLs are produced exclusively by some slow growing mycobacteria (e.g. *M. tuberculosis, M. kansasii, M. marinum, M. ulcerans *and *M. leprae*) [29]. Our phylogenetic screen detected Rv2949c, the chorismate-formate lyase responsible for production of the phenol ring [30], as an HGT in the ancestor of these bacteria (List B). We also detected other HGT in this locus, notably Rv2962c and Rv2958c (List B) and Rv2959c (List C), responsible for the addition of different sugars to produce the *M. tuberculosis *form of PGL [31]. Lastly, the methyltransferase Rv2959c, implicated in the O-methylation of one of the hydroxyl groups of the rhamnosyl residue of *M. tuberculosis *PGL [32], was also detected as a foreign gene (List C). As PGL is an important *M. tuberculosis *virulence factor [33] and the enzymes responsible for building from DIM to PGL to the *M. tuberculosis *form of PGL have been horizontally acquired, this locus provides a clear illustration of how HGT has contributed to the step-wise evolution of virulent *M. tuberculosis*. A similar scenario applies for the LOS locus. This locus has been implicated in the production of different variants of these highly polar glycolipids in *M. marinum *[34], although the complete locus is not conserved in all *M. tuberculosis *strains [35]. Again, our phylogenetic analysis uncovered evidence of HGT in the form of transferases that affect the species-specificities of LOS products. Moreover, our study has also shown a number of horizontally acquired transferases outside of these two loci. As the acquisition of such enzymes can serve to modify ancestral compounds, a future challenge will be to identify the substrates of these enzymes and determine the role of the newly synthesized compounds in *M. tuberculosis *pathogenicity.

Beyond biochemical modification of mycobacterial virulence factors, genes acquired through HGT have potentially mediated the transition from an environmental niche to intracellular life within a complex eukaryotic host. As a first example, two loci are predicted to mediate adaptation to anaerobic conditions. The *hyc *operon shows homology with the formate hydrogenlyase operon from enterobacteria, implicated in formate fermentation by transforming formate into CO_2 _and H_2 _[36]. Three genes of this operon were predicted to be essential by TraSH analysis (2 *in vitro *and one for macrophage infection), therefore this operon has an important role in *M. tuberculosis *metabolism that merits further study. The second locus shows homology with the fumarate reductase operon, which is required for anaerobic respiration using fumarate as the terminal electron acceptor [37]. As both of these operons are up-regulated during oxygen starvation [38,39], it can be hypothesized that the acquisition of these genes provided a selective advantage for residence in anaerobic conditions, as encountered within a granuloma [40]. A second example is the acquisition of genes Rv3373 to Rv3378c. Two of these genes have been shown to be essential during infection of mice (Rv3375 and Rv3376) [23]. Curiously, Rv3377c and Rv3378c encode proteins implicated in the production of a compound (tuberculosinol) with a undefined function, never documented in bacteria [41]. These two genes were also identified in a genetic screen for inhibitors of macrophage phagosome acidification [42], suggesting a role for this compound in modulating the intracellular environment.

## Conclusion

We have demonstrated step-wise HGT during the genesis of *M. tuberculosis *with evidence that this process has mediated the acquisition of established virulence factors. Our original approach has permitted the detection of 'old' HGT which may have increased the pathogenic potential of slow-growing mycobacteria along with *M. tuberculosis*-specific HGT which may have permitted its adaptation to the human lung. The examples provided in this manuscript, along with other published examples of HGT [8,43,44] present opportunities to formally test this, for example, by analyzing for gain-of-function when genetically engineering insertion events into non-tuberculous mycobacteria. Additionally, as our model and software permit the genomic analysis of different bacteria, it will also be possible to determine the degree to which HGT has contributed to the step-wise evolution of other mycobacterial and non-mycobacterial pathogens.

## Authors' contributions

FV participated in the conception of the study, in the program's designing and testing, carried out analyzes of the data and drafted the manuscript. DP participated to the program's designing and testing. CT carried out the MLSA analyzes. MB conceived of the study, and participated in its design and coordination and helped to draft the manuscript. All authors read and approved the final manuscript.

## Supplementary Material

Additional file 1 **Additional Figures S1 to S5**. The file provided different supplementary Figures: S1: Frequency histogram of the distribution of blast scores; S2: Screenshot of mycoHIT; S3: Table representing the number of amino acid differences among the 20 housekeeping genes; S4: Graphic representation of HGT clusters; S5: Number and proportion of genes from Lists A, B, C and D presenting specified characteristics. Click here for file

Additional file 2 **Potential *M. tuberculosis *genes deletion**. The file provided the protocol and the results to detect genes potentially deleted from the *M. tuberculosis *ancestor at each strata of its evolution (Lists Bd, Cd, Dd). Click here for file
