# Supplementary material for: Phylogenetic detection of horizontal gene transfer during the step-wise genesis of Mycobacterium tuberculosis
Source: BMC Evol Biol. 2009 Aug 10;9:196. doi: 10.1186/1471-2148-9-196 (PMC3087520; doi:10.1186/1471-2148-9-196)
Supplement: Additional file 1 — Additional Figures S1 to S5. The file provided different supplementary Figures: S1: Frequency histogram of the distribution of blast scores; S2: Screenshot of mycoHIT; S3: Table representing the number of amino acid differences among the 20 housekeeping genes; S4: Graphic representation of HGT clusters; S5: Number and proportion of genes from Lists A, B, C and D presenting specified characteristics. [file 1471-2148-9-196-S1.ppt]

## Slide 1
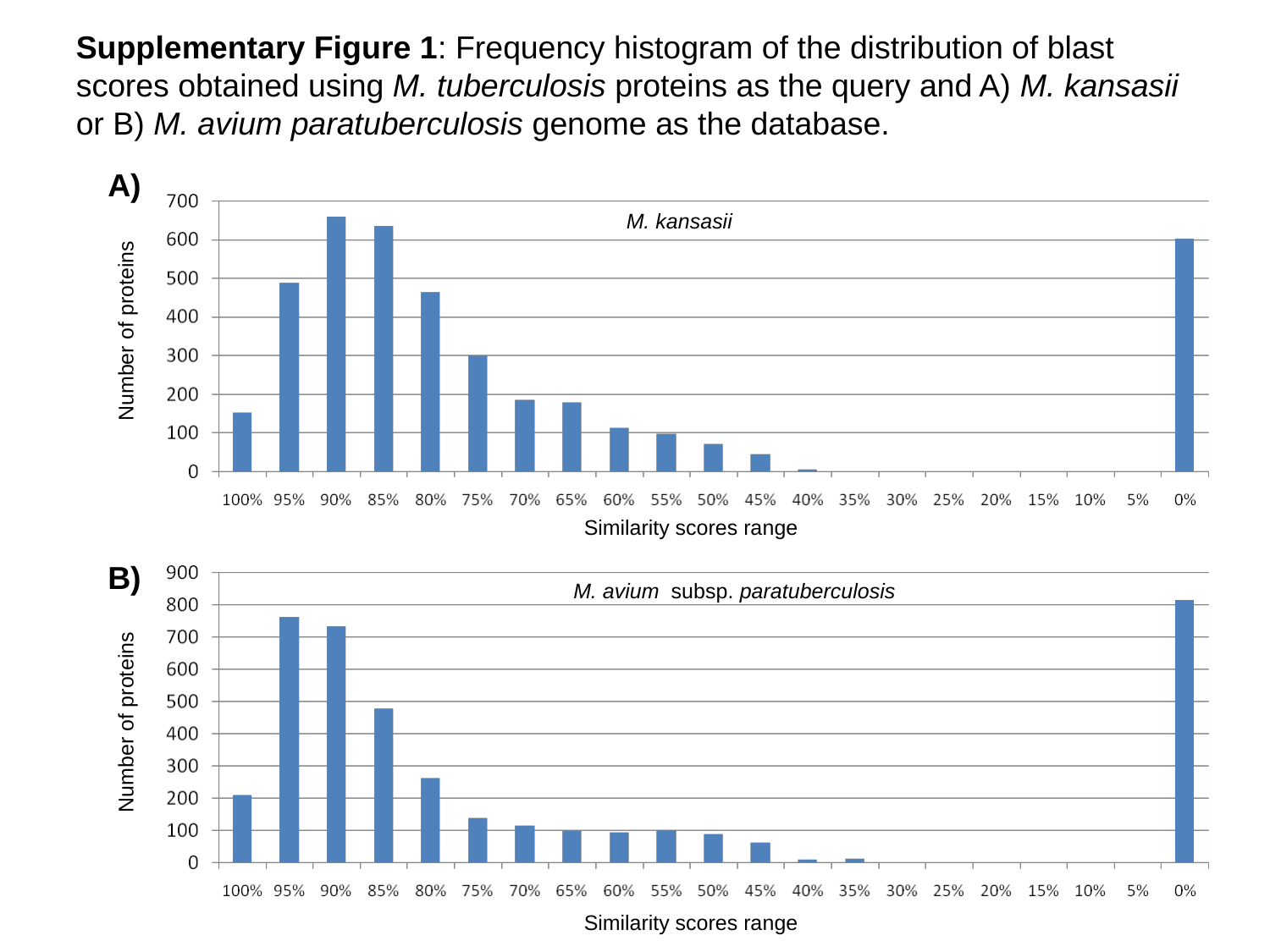

Supplementary Figure 1: Frequency histogram of the distribution of blast scores obtained using M. tuberculosis proteins as the query and A) M. kansasii or B) M. avium paratuberculosis genome as the database.
A)
M. kansasii
Number of proteins
Similarity scores range
B)
M. avium subsp. paratuberculosis
Number of proteins
Similarity scores range

## Slide 2
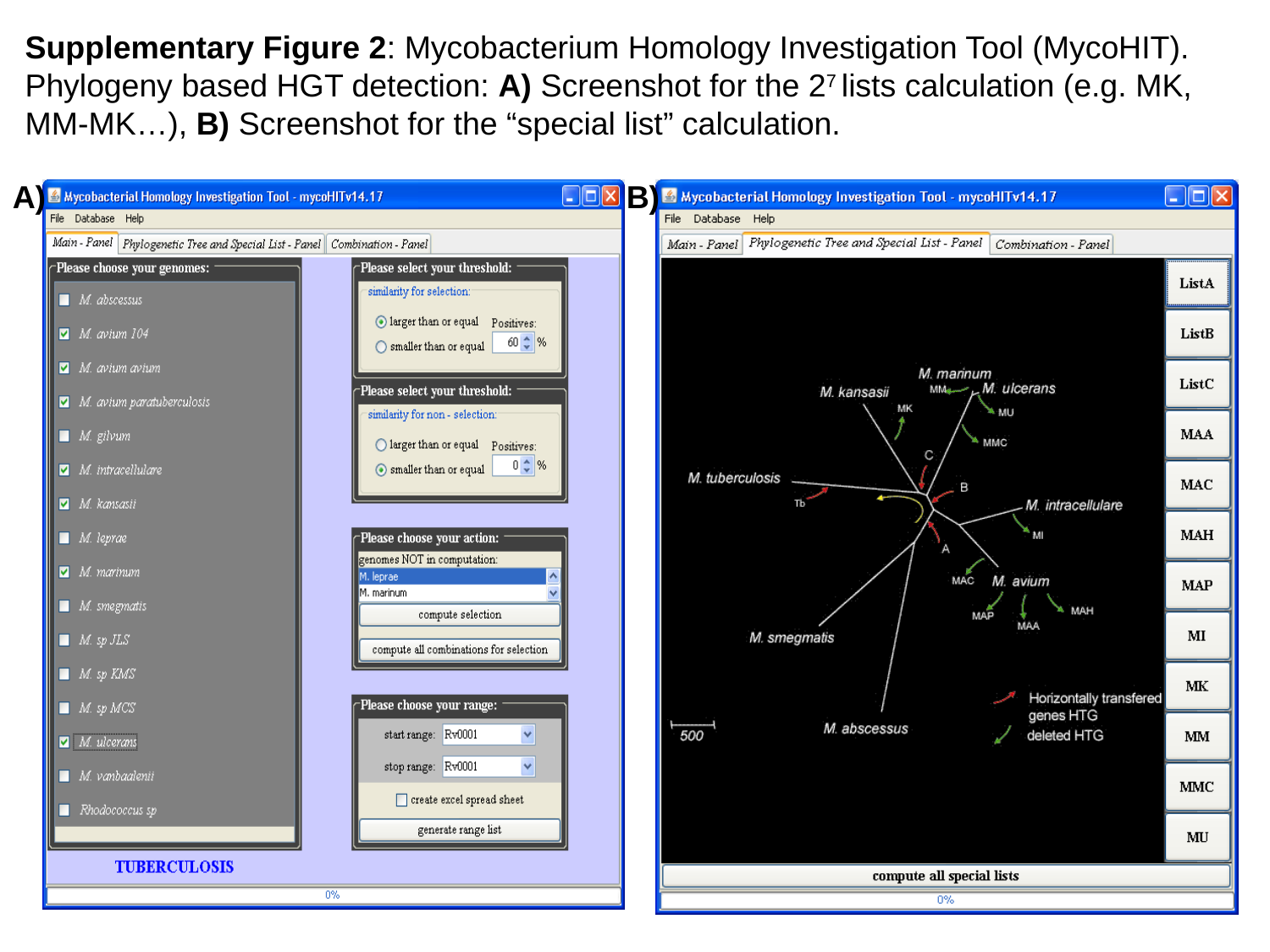

Supplementary Figure 2: Mycobacterium Homology Investigation Tool (MycoHIT). Phylogeny based HGT detection: A) Screenshot for the 27 lists calculation (e.g. MK, MM-MK…), B) Screenshot for the “special list” calculation.
A)
B)

## Slide 3
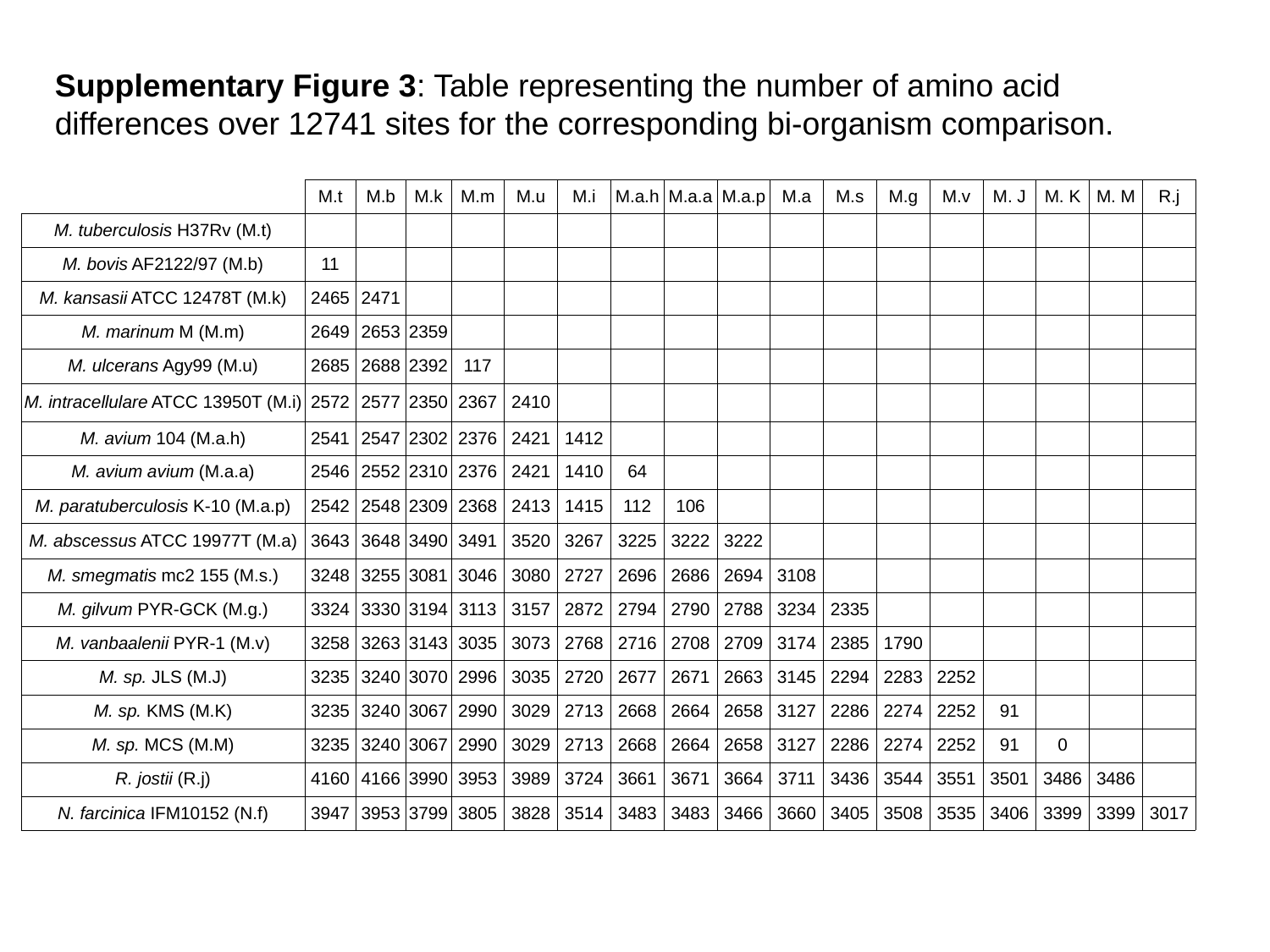

Supplementary Figure 3: Table representing the number of amino acid differences over 12741 sites for the corresponding bi-organism comparison.
| | M.t | M.b | M.k | M.m | M.u | M.i | M.a.h | M.a.a | M.a.p | M.a | M.s | M.g | M.v | M. J | M. K | M. M | R.j |
| --- | --- | --- | --- | --- | --- | --- | --- | --- | --- | --- | --- | --- | --- | --- | --- | --- | --- |
| M. tuberculosis H37Rv (M.t) | | | | | | | | | | | | | | | | | |
| M. bovis AF2122/97 (M.b) | 11 | | | | | | | | | | | | | | | | |
| M. kansasii ATCC 12478T (M.k) | 2465 | 2471 | | | | | | | | | | | | | | | |
| M. marinum M (M.m) | 2649 | 2653 | 2359 | | | | | | | | | | | | | | |
| M. ulcerans Agy99 (M.u) | 2685 | 2688 | 2392 | 117 | | | | | | | | | | | | | |
| M. intracellulare ATCC 13950T (M.i) | 2572 | 2577 | 2350 | 2367 | 2410 | | | | | | | | | | | | |
| M. avium 104 (M.a.h) | 2541 | 2547 | 2302 | 2376 | 2421 | 1412 | | | | | | | | | | | |
| M. avium avium (M.a.a) | 2546 | 2552 | 2310 | 2376 | 2421 | 1410 | 64 | | | | | | | | | | |
| M. paratuberculosis K-10 (M.a.p) | 2542 | 2548 | 2309 | 2368 | 2413 | 1415 | 112 | 106 | | | | | | | | | |
| M. abscessus ATCC 19977T (M.a) | 3643 | 3648 | 3490 | 3491 | 3520 | 3267 | 3225 | 3222 | 3222 | | | | | | | | |
| M. smegmatis mc2 155 (M.s.) | 3248 | 3255 | 3081 | 3046 | 3080 | 2727 | 2696 | 2686 | 2694 | 3108 | | | | | | | |
| M. gilvum PYR-GCK (M.g.) | 3324 | 3330 | 3194 | 3113 | 3157 | 2872 | 2794 | 2790 | 2788 | 3234 | 2335 | | | | | | |
| M. vanbaalenii PYR-1 (M.v) | 3258 | 3263 | 3143 | 3035 | 3073 | 2768 | 2716 | 2708 | 2709 | 3174 | 2385 | 1790 | | | | | |
| M. sp. JLS (M.J) | 3235 | 3240 | 3070 | 2996 | 3035 | 2720 | 2677 | 2671 | 2663 | 3145 | 2294 | 2283 | 2252 | | | | |
| M. sp. KMS (M.K) | 3235 | 3240 | 3067 | 2990 | 3029 | 2713 | 2668 | 2664 | 2658 | 3127 | 2286 | 2274 | 2252 | 91 | | | |
| M. sp. MCS (M.M) | 3235 | 3240 | 3067 | 2990 | 3029 | 2713 | 2668 | 2664 | 2658 | 3127 | 2286 | 2274 | 2252 | 91 | 0 | | |
| R. jostii (R.j) | 4160 | 4166 | 3990 | 3953 | 3989 | 3724 | 3661 | 3671 | 3664 | 3711 | 3436 | 3544 | 3551 | 3501 | 3486 | 3486 | |
| N. farcinica IFM10152 (N.f) | 3947 | 3953 | 3799 | 3805 | 3828 | 3514 | 3483 | 3483 | 3466 | 3660 | 3405 | 3508 | 3535 | 3406 | 3399 | 3399 | 3017 |

## Slide 4
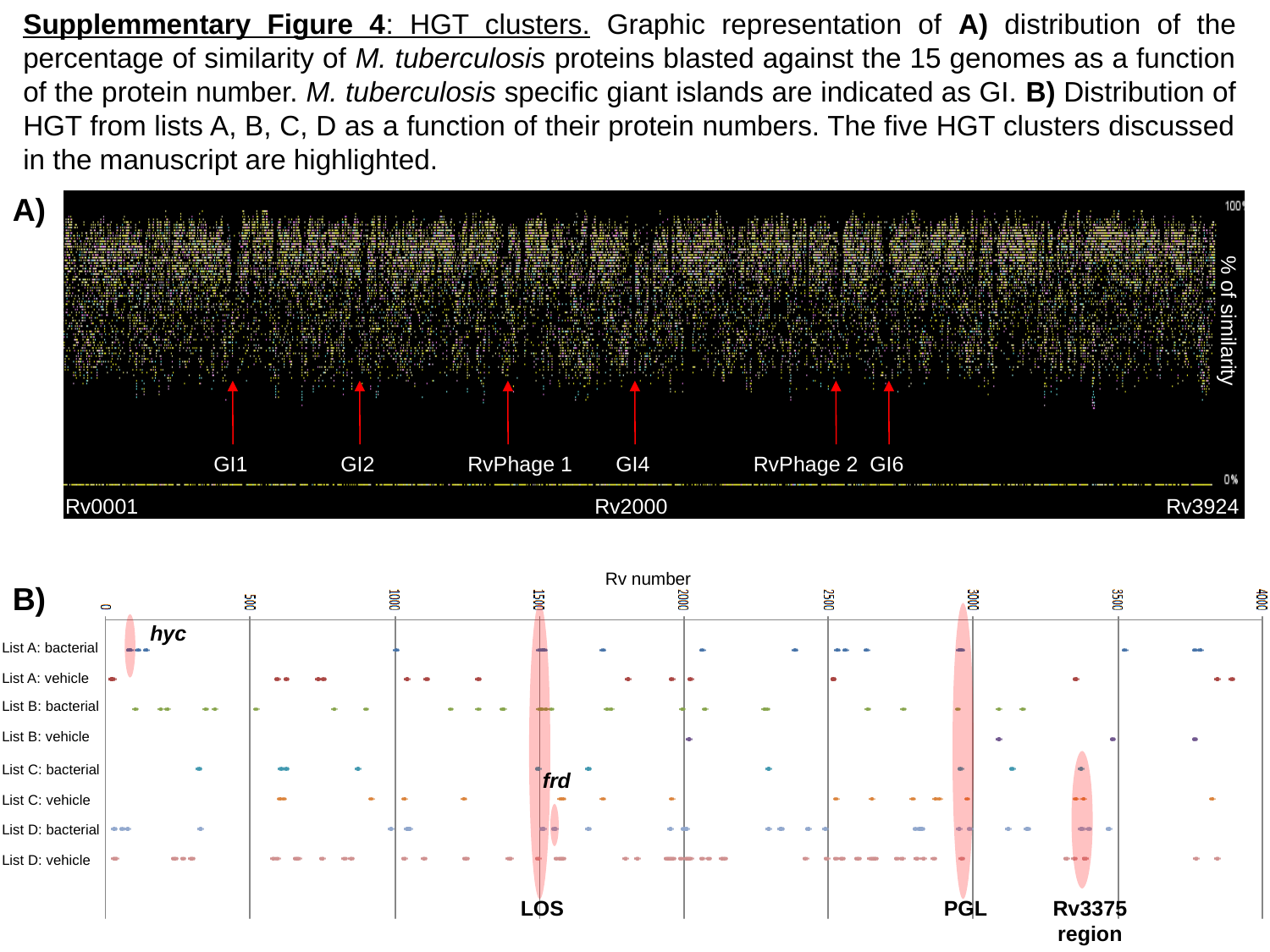

Supplemmentary Figure 4: HGT clusters. Graphic representation of A) distribution of the percentage of similarity of M. tuberculosis proteins blasted against the 15 genomes as a function of the protein number. M. tuberculosis specific giant islands are indicated as GI. B) Distribution of HGT from lists A, B, C, D as a function of their protein numbers. The five HGT clusters discussed in the manuscript are highlighted.
A)
% of similarity
GI1
GI2
RvPhage 1
GI4
RvPhage 2
GI6
Rv0001
Rv2000
Rv3924
Rv number
B)
hyc
List A: bacterial
List A: vehicle
List B: bacterial
List B: vehicle
List C: bacterial
frd
List C: vehicle
List D: bacterial
List D: vehicle
LOS
PGL
Rv3375
region

## Slide 5
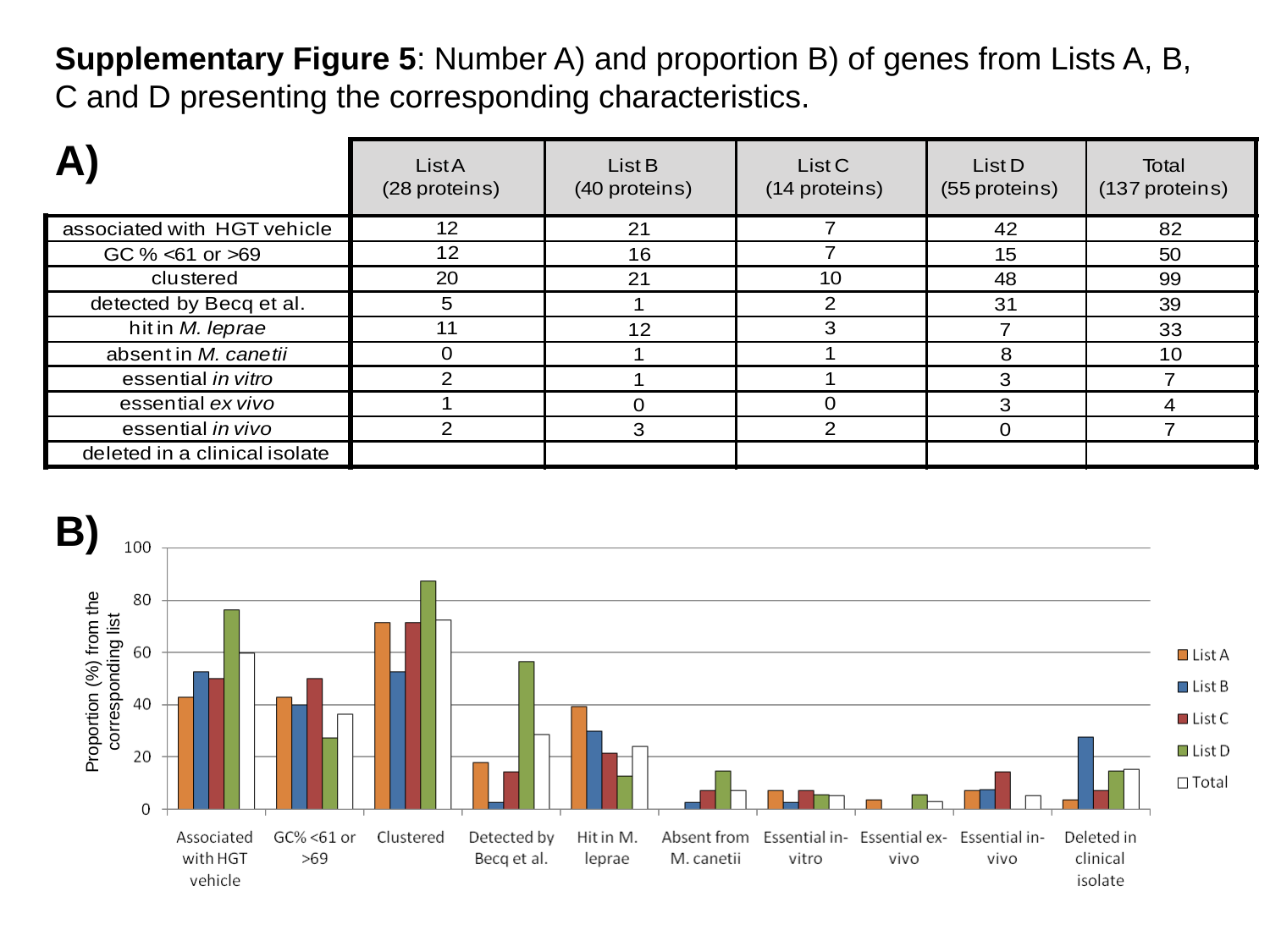

Supplementary Figure 5: Number A) and proportion B) of genes from Lists A, B, C and D presenting the corresponding characteristics.
A)
B)
Proportion (%) from the corresponding list
